# A Delay-Cell-Controlled VCO Design for Unipolar Single-Gate Enhancement-Mode TFT Technologies

**DOI:** 10.3390/mi14010032

**Published:** 2022-12-23

**Authors:** Bin Li, Siwei Wei, Mingjian Zhao, Rongsheng Chen, Zhaohui Wu, Yuming Xu

**Affiliations:** 1 School of Microelectronics, South China University of Technology, Guangzhou 510640, China; 2 Pazhou Laboratory, Guangzhou 510330, China; 3 Huawei Technologies Company, Ltd., Shenzhen 518129, China

**Keywords:** thin film transistors, voltage control oscillator, ring oscillator

## Abstract

This work outperforms the previous literatures by proposing a delay-cell-controlled voltage control oscillator (VCO) design for common unipolar, single-gate, and enhancement-mode thin-film transistor (TFT) technologies. A design example with InZnO TFTs is simulated to verify the proposed design. The design example has a 500 μW power consumption, 0.7 mm^2^ area, 3.8 kHz–8 kHz output frequency range, 600 Hz/V tuning sensitivity, and 4% maximum linear error. This design may have the potential to be used for flexible, low cost, and moderate speed sensor readout interfaces.

## 1. Introduction

Thin-film transistor (TFT) circuits have made great progress in recent years. Complicated circuits and systems including ARM processers [1], analog frontends and conversion circuits [2], and wireless communication systems [3] have been reported. Voltage control oscillators (VCOs) are an integral part of many electronic systems. Table 1 reviews the existing TFT-based VCO designs [4,5,6,7,8,9,10]. This work focuses on the digital ring oscillator (RO) architecture with delay-cell-control scheme since it has the advantages of the simple and compact structure, the high input impedance, the digital output that can be processed directly using digital circuitry without shaping, and it does not require analogue blocks such as high-performance operation amplifiers that are difficult to achieve with current TFT technologies. The delay-cell-control VCO design that was proposed for the first time in [6], however, was designed for the uncommon dual-gate and depletion-mode TFT device. This work solves this issue by presenting another design for the common TFT devices, i.e., unipolar (non-complementary), single-gate, and enhancement-mode TFTs.

## 2. Device

**Figure 1:**
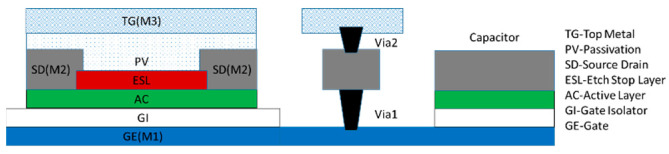
The IZO TFT technology used.

The design example was based on our 10 μm channel length, n-type, etch stop layer (ESL), and InZnO (IZO) TFTs. The device has a bottom gate and top contact structure on glass substrate as shown in Figure 1. Three metal layers are provided: the gate metal (M1), the source/drain metal (M2), and the top metal (M3) for further connection. The gate metal is a 200 nm thick molybdenum (Mo) layer (M1). The gate insulator (GI) is two stacked layers of SiN_x_/SiO_2_ with 200 nm/50 nm. The active layer is a 30 nm thick IZO. Source and drain electrodes are 200 nm thick Mo layers (M2). A 300 nm SiO_2_ is formed as a passivation layer (PV) for protecting the TFT devices. A layer of Ni serves as the contact electrode (M3). The typical threshold voltage (V_th_), mobility, and subthreshold slope are 3 V, 10.5 cm^2^V^−1^s^−1^, and 110 mV/dec, respectively. Because of the bottom gate structure, overlapping capacitance per unit channel width is 1.41 nF/m. On-chip capacitors with 19 nF/cm^2^ are also available by using the M1 and M2 as two plates and the gate oxide as an insulating layer. More details about the TFT device technology can be found elsewhere [11].

**Figure 2:**
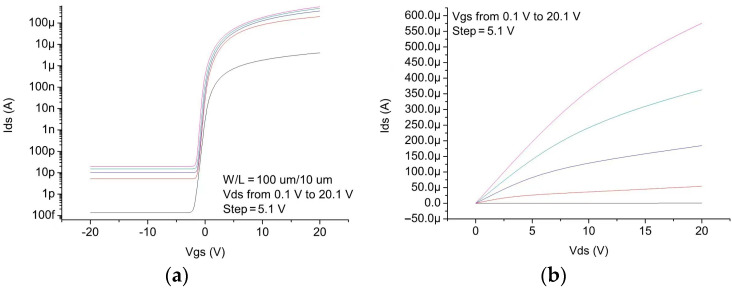
Simulated (**a**) transfer and (**b**) output curves of the IZO TFT device.

Characteristics of the TFT devices used are calibrated with experimental data [11]. An Hspice Level = 62 RPI Poly Si TFT Model is established using parameter extraction to fit the measured device characteristics. Figure 2 shows the simulated transfer and output curves of the device.

## 3. Circuit Design

**Figure 3:**
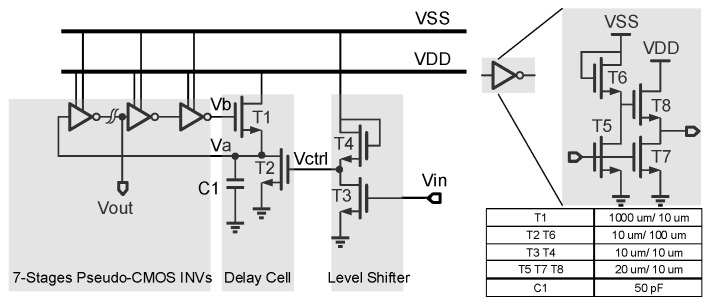
Schematic of the proposed TFT VCO.

**Figure 4:**
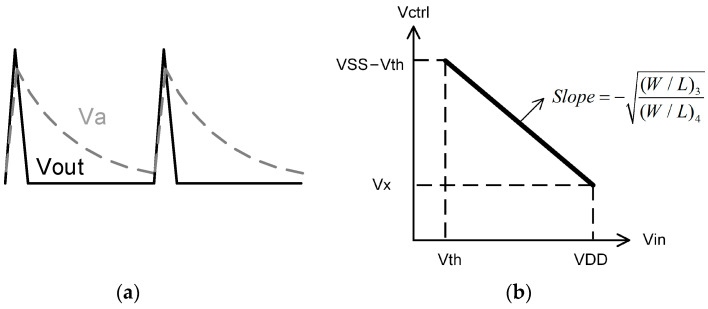
(**a**) Qualitative sketch of the main waveforms of the proposed VCO, and (**b**) voltage transfer curve of the level shifter.

Figure 3 shows the schematic of the proposed VCO. It consists of a RO and a level shifter, while the RO consists of an odd number of inverters and a non-inverting delay cell. It generates self-oscillation with the frequency adjusted using a voltage control resistor realized through T2. The waveform of the delay cell output (node V_a_) is shaped using the inverter chains and becomes a series of pulses at the output node V_out_. Typical waveforms of the circuit nodes are shown in Figure 4a.

The delay cell consists of T1, T2, and C_1_. The rising time constant of the delay cell is proportional to R_1_C_1_ while C_1_ is charging through T1, and the falling time constant of the delay cell is proportional to R_2_C_1_ while C_1_ is discharging through T2, whereas R_1_ and R_2_ are equivalent resistances of T1 and T2, respectively. T1 and T2 are both biased in the deep linear region so that they can act as resistors. R_2_ is a voltage control resistor controlled using the gate voltage of T2, V_ctrl_, thus the oscillation period can be controlled using V_ctrl_. If the rising time and delay of the inverter chain are both much smaller than the falling time, the oscillation frequency can be proportional to V_crtl_, thus (W/L)_1_ is designed to be much greater than (W/L)_2_, to make R_2_ much greater than R_1_. Also, a relatively large capacitance is chosen. The oscillation period can be given using

(1) $$T_{\mathrm{OSC}}{=T}_{\mathrm{INV}}{+T}_{R}{+T}_{F}\;\approx {\;T}_{F}\;\propto {\;R}_{2}C_{1}$$

where T_INV_ is the inverter chain delay, T_R_ is the rising time of the delay cell, T_F_ is the falling time of the delay cell, and R_2_ is the equivalent resistance of T2.

The pull-down transistor T2 is designed to be biased in the deep linear region so that it can act as a voltage control resistor whose resistance is given by

(2) $$R_{2}=\frac{1}{{\mu C}_{\mathrm{OX}}{\left(W/L\right)}_{2}{(V}_{\mathrm{ctrl}}-V_{\mathrm{th}})}$$

Combining (1) and (2), the oscillation frequency is given using

(3) $$f_{\mathrm{OSC}}\;\propto \;\frac{{\mu C}_{\mathrm{OX}}{\left(W/L\right)}_{2}{(V}_{\mathrm{ctrl}}-V_{\mathrm{th}})}{C_{1}}$$

To ensure T2 in the linear region, its gate voltage V_ctrl_ should be higher than VDD-V_th_. However, the input signal Vin ranges from GND to VDD, which cannot meet the requirement. Thus, a level shifter is designed to boost the V_in_ to above VDD-V_th_.

The level shifter is actually a diode-load inverter or amplifier that is powered using VSS. Figure 4b gives the voltage transfer curve of the level shifter. When V_in_ increases from V_th_ to VDD, V_ctrl_ linearly decreases from VSS−V_th_ to V_x_. The slope of the transfer curve equals to $-\sqrt{\frac{{\left(W/L\right)}_{3}}{{\left(W/L\right)}_{4}}}$. Thus, the transfer function of the level shifter is given using

(4) $$V_{\mathrm{ctrl}}=-\sqrt{\frac{{\left(W/L\right)}_{3}}{{\left(W/L\right)}_{4}}}{(V}_{\mathrm{in}}-V_{\mathrm{th}})+\mathrm{VSS}-V_{\mathrm{th}}$$

(5) $$V_{\mathrm{in}}\;\geq {\;V}_{\mathrm{th}}$$

To make sure that T3 is in the saturation region and T2 is in the linear region, V_x_ must be higher than VDD−V_th_. Therefore, the sizes of T3 and T4 should satisfy

(6) $$\frac{\mathrm{VSS}-\mathrm{VDD}}{\mathrm{VDD}-\mathrm{Vth}}\;\geq \;\sqrt{\frac{{\left(W/L\right)}_{3}}{{\left(W/L\right)}_{4}}}$$

In this design, the VSS is set to be 2VDD [12], and T3 and T4 are designed to have the same size. Since its gain remains constant over the entire input range, the level shifter does not introduce linear errors into the VCO. Signals will be inverted after passing through the level shifter, so the input voltage and output frequency are ultimately inversely proportional.

Combining (3) and (4), we can give the final f-V characteristic using

(7) $$f_{\mathrm{OSC}}\;\propto -\frac{{\mu C}_{\mathrm{OX}}{\left(W/L\right)}_{2}}{C_{1}}[\sqrt{\frac{{\left(W/L\right)}_{3}}{{\left(W/L\right)}_{4}}}\left(V_{\mathrm{in}}-V_{\mathrm{th}}\right){+2V}_{\mathrm{th}}-\mathrm{VSS}]$$

The above formula can be further described as

(8) $$f_{\mathrm{OSC}}{=K}_{\mathrm{VCO}}\left(V_{\mathrm{in}}-V_{\mathrm{th}}\right)+\frac{{2V}_{\mathrm{th}}-\mathrm{VSS}}{\sqrt{\frac{{\left(W/L\right)}_{3}}{{\left(W/L\right)}_{4}}}}K_{\mathrm{VCO}}$$

(9) $$K_{\mathrm{VCO}}=-K\frac{{\mu C}_{\mathrm{OX}}{\left(W/L\right)}_{2}}{C_{1}}\sqrt{\frac{{\left(W/L\right)}_{3}}{{\left(W/L\right)}_{4}}}$$

where K_VCO_ represents the tuning sensitivity and K is a scale constant.

In summary, the properties of the proposed VCO are described by (8) and (9), with the constraints described by (5) and (6).

One of the sources of the nonlinearity of the proposed VCO is the delay of the inverter chains and the rising time of the delay cell, as described in (1). Therefore, a large value of C1 and a small size of T2 are desired to achieve a high linearity. However, according to (8) and (9), this will reduce the output frequencies as well as the KVCO. A tradeoff between linearity, oscillation frequencies, and sensitivity is found in the proposed design. Another source of the nonlinearity derives from the deviation between the actual device characteristics and the square-law model that makes T2 not behave as an ideal voltage-controlled resistor.

## 4. Simulation Results and Discussion

**Figure 5:**
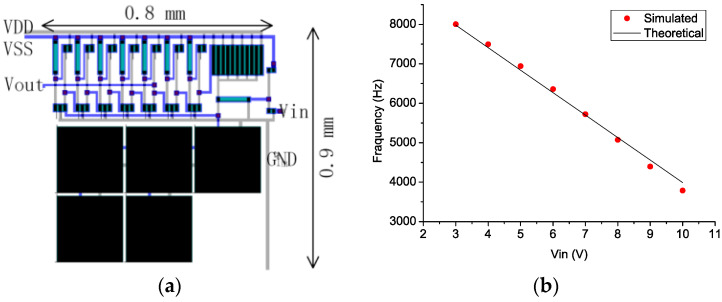
(**a**) Layout of the proposed VCO, and (**b**) simulated and theoretical V-f characteristics of the proposed VCO.

Figure 5a shows the layout of the proposed VCO. The VCO occupies 0.7 mm^2^ area fully on-chip and consumes 500 μW power.

The simulations were performed under VDD = 10 V and VSS = 20 V. The reason for choosing these supply voltages is the relatively high V_th_ (~3 V) of our TFT devices. VCO outputs under different input voltages that range from V_th_ to VDD were captured.

Figure 5b shows the simulated voltage versus frequency curve of the VCO. The output frequency decreases from 8 kHz to 3.8 kHz as Vin increases from 3 V to 10 V.

Theoretical curves that are calculated (8) using K_VCO_ of −570, VSS of 20 V, and V_th_ of 3V, are also shown in Figure 5b. It is found that the theoretical curve fits well with the simulated curve. This means that the theoretical analysis does provide an accurate prediction of circuit behavior. Differences between simulated and theoretical characteristics result from the deviation between the actual device characteristics and the square-law model.

**Figure 6:**
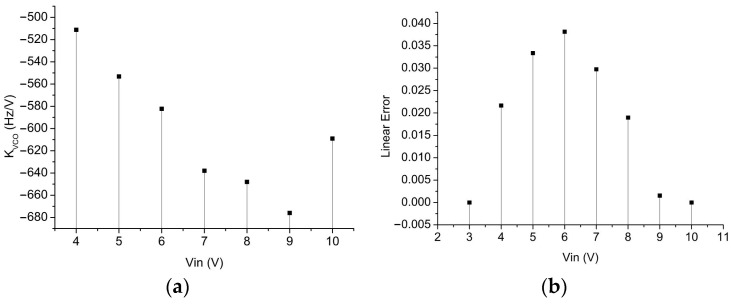
Simulated (**a**) K_VCO_ and (**b**) linear error of the proposed VCO.

Figure 6a shows the voltage versus K_VCO_ curve of the VCO. The value of K_VCO_ ranges from 510 Hz/V to 680 Hz/V. The average value of K_VCO_ is 600 Hz/V. Figure 6b shows the linear error of the VCO that was normalized using the output frequency range. The maximum linear error is 4% and appears at V_in_ = 6 V. The linear error is calculated as the difference between the measured f-V curve and the linear fit through its extremes.

Table 2 summarizes the performance of the proposed VCO and compares it to the state-of-the-art counterparts. This work outperforms [6] by proposing a design suitable for the single-gate and enhancement TFT devices. Moderate output frequencies and tuning sensitivity are achieved, due to the inherent speed–linearity trade-off of the proposed design. In addition, excellent circuit integration and power consumption are performed, thanks to the compact and all-digital architecture. According to these results, it is suggested that the proposed design may have the potential to be used for low-cost, moderate speed applications such as voltage-to-frequency converters for flexible sensor interfaces.

## 5. Conclusions

A delay-controlled VCO design for unipolar, single-gate, and enhancement-mode TFT technologies has been proposed. Theoretical analysis and design guidelines have been given. A design example based on IZO TFTs has been proposed to verify the design. It is found that the simulation results of the design example fit well with the theoretical analysis, showing moderate speed and linearity, excellent integration, and power consumption compared to the literatures.

## Figures and Tables

**Table 1:** Summary of the existing TFT VCO designs.

	Simplified Schematic	Input Impedance	Output Waveform
Digital RO with VDD control [4,5]	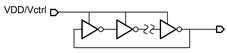	Low	Digital square wave 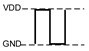
Digital RO with delay-cell-control [6]	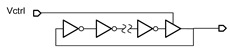	High	Digital pulse 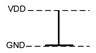
Analog RO with tail-current source-control [7]	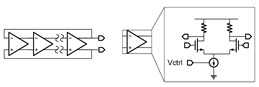	High	Analog sinewave 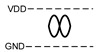
Bistable oscillator [8]	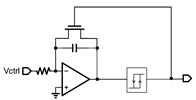	Moderate ^2^	Digital pulse 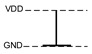
Relaxation oscillator [9]	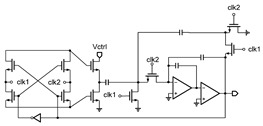	Low	Digital square wave 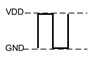
LC oscillator [10] ^1^	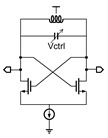	High	Analog sinewave 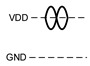

^1^ This design with active inductors and light-sensing scheme is shown, while the common LC tank VCO design with the capacitor control scheme is shown in the simplified schematic. ^2^ The design depends on the value of the input resistor.

**Table 2:** Summary and comparison table.

	[4]	[5] ^1,2^	[6] ^2^	[7]	[8] ^1^	[9] ^1^	This Work ^1^
TFT type	Organic unipolar single-gate enhancement	Oxide unipolar single-gate enhancement	Organic unipolar dual-gate depletion	Oxide unipolar single-gate enhancement	Oxide unipolar single-gate enhancement	Oxide unipolar single-gate enhancement	Oxide unipolar single-gate enhancement
VCO architecture	Digital RO with VDD control	Digital RO with VDD Control	Digital RO with delay-cell-control	Analog RO with tail-current source-control	Bi-stable oscillator	Relaxation oscillator	Digital RO with delay-cell-control
Supply (V)	−20–−15	6–14	20	15	VDD = 6 V VSS = 8 V	±5	VDD = 10 V VSS = 20 V
Power (μW)	150–450 ^3^	100–1000 ^3^	6	1500	109	1300	500
Area (mm^2^)	-	0.3 ^3^	1 ^3^	3.37	-	-	0.7
Output frequency range (Hz)	1.3 k–2.1 k ^3^	100 k–200 k ^3^	4—38	111 k–171 k	1 k–2 k	400–550 ^3^	3.8 k–8 k
Input voltage range (V)	−20–−15	6–14	0–20	2–15	1–2	−5– +5	3–10
Average tuning sensitivity (Hz/V)	160	12.5 k	1.7	4.6 k	1 k	15	600
Max linear error	0.011 ^3^	0.01–0.13 ^3^	0.016	0.04	0.016	Large	0.04

^1^ Simulation results. ^2^ The results consider only the VCO part. ^3^ Typical results estimated from figures and tables in references.

## Data Availability

Not applicable.
